# Nutritional and environmental assessment of school menus served, consumed and wasted in primary schools in Spain: a comparison of public and charter schools

**DOI:** 10.1017/S1368980025101158

**Published:** 2025-09-19

**Authors:** Naiara Martinez-Perez, Rocío Barrena-Barbadillo, Iñaki Irastorza-Terradillos

**Affiliations:** 1 Department of Nursing I, University of the Basque Country UPV/EHU, 48940 Leioa, Spain; 2 BIOMICs Research Group, Microfluidics & BIOMICs Cluster UPV/EHU, Lascaray Research Centre, University of the Basque Country UPV/EHU, 01006 Vitoria-Gasteiz, Spain; 3 Biocruces Bizkaia Health Research Institute, 48903 Barakaldo, Spain; 4 Department of Pediatrics, BioBizkaia, Cruces University Hospital, University of the Basque Country UPV/EHU, 48903 Barakaldo Spain

**Keywords:** Plate waste, School lunch, Nutritional adequacy, Carbon footprint

## Abstract

**Objective:**

To assess the nutritional composition, adequacy and environmental impact of menus served, consumed and wasted by 11–12-year-old students in public and charter schools in northern Spain.

**Design:**

A cross-sectional observational study (2017–2018) involving photographing menus before and after consumption, visual portion size estimation using a validated photographic catalogue and food waste assessment via the quarter-waste visual method. Nutritional composition was analysed using food composition databases and greenhouse gas emissions using life cycle assessment data.

**Setting:**

Ten primary schools (five public and five charter) in northern Spain.

**Participants:**

1000 school menus for students aged 11–12 years.

**Results:**

Menus served exceeded energy recommendations (791·5 (sd 176·7) kcal) and were high in fat (39·7 (sd 13·4) g), protein (29·7 (sd 10·0) g) and Na (980·4 (sd 302·2) mg) but low in carbohydrates (74·7 (sd 18·1) g), fibre (8·8 (sd 3·7) g) and several micronutrients. Food waste averaged 140·5 g per menu, mainly vegetables and fruit, leading to nutrient losses, particularly in fibre, vitamins A and C and Fe. The carbon footprint of menus averaged 1·489 kg CO_2_-eq, primarily from meat and fish, with waste contributing 0·298 kg CO_2_-eq. Public schools served more nutrient-dense food but had higher waste (public 151·5 (sd 112·3) g *v*. charter 129·5 (sd 86·3) g, *P* < 0·001); charter schools served more energy-dense food, with higher Na and fat (*P* < 0·001).

**Conclusions:**

Menus showed nutritional imbalances, with excessive energy and Na and insufficient fibre and several micronutrients. Food waste worsened dietary adequacy while increasing environmental impact. Public schools offered more nutrient-rich food but faced greater waste compared with charter schools. Institutional differences suggest the need for tailored strategies to enhance both nutritional quality and sustainability.

Childhood is a critical period for growth and development, during which adequate nutrition is essential to promote health and prevent both short- and long-term problems. A balanced diet contributes to physical and cognitive development^(1)^ and helps prevent diseases such as obesity, micronutrient deficiencies and other diet-related chronic diseases^(2)^. Schools, where children spend a large part of their day, are key to promoting healthy eating habits. For many children, school canteens provide approximately one-third of daily energy intake^(3)^. In Spain, around half of primary school students eat lunch at school canteens^(4)^, offering a unique opportunity to foster healthy habits in a supervised setting.

School menus should be nutritionally balanced, encouraging the consumption of fruits, vegetables and legumes while limiting ultra-processed foods high in sugars and saturated fats^(5)^. Additionally, menus should appeal to children’s taste and presentation preferences, which strongly influence acceptance and consumption^(5,6)^. However, studies show that school meals often fail to meet nutritional recommendations, providing insufficient levels of carbohydrates, fibre and vitamins while being excessive in fats and proteins^(7–9)^. This is worsened by the large amount of food waste^(7,10)^, especially of nutrient-rich items such as vegetables, fruits and fish^(9,11)^. Such waste suggests that children may not fully benefit from the intended nutritional value of school meals^(12)^, hindering efforts to promote healthy eating.

Beyond nutrition, food waste has significant social and environmental implications. The food system contributes 20–40 % of global greenhouse gas emissions^(13)^, with animal-based foods being the most carbon-intensive^(14)^. Wasted food further exacerbates this burden by wasting the resources used in its production. Up to one-third of global food is lost or wasted, with food services contributing 26 %^(15)^. Reducing food waste, especially of high-emission items like meat and dairy products, is crucial to minimising the environmental footprint of school meals.

While previous research has examined either school meals nutritional quality or food waste, few studies have addressed both aspects alongside environmental impact, particularly across different school types (e.g. public *v*. charter)^(7,9,10)^. For instance, Liz Martins *et al*. (2021) analysed nutritional adequacy and plate waste in Portuguese schools without addressing environmental impact^(9)^, while Biasini *et al*. (2024) assessed the carbon footprint (CF) in Italian schools without comparing school types^(16)^. This study addresses these gaps by combining nutritional and environmental metrics across public and charter schools, providing insights into how institutional frameworks influence dietary outcomes and sustainability. This approach is essential for designing school meal policies that align with health and environmental goals.

Thus, the aim of this study is twofold: (i) to analyse the nutritional composition and adequacy of menus served and consumed by 11- and 12-year-old students in public and charter primary schools in northern Spain and (ii) to assess the CF of food served and wasted in these schools. By examining food served, consumed and wasted, this study seeks to identify opportunities to improve both the nutritional quality and environmental sustainability of school menus, while reducing food waste and improving student acceptance.

## Material and methods

### Study design

A cross-sectional observational study was conducted between 2017 and 2018 in public and charter primary schools in Bizkaia, Spain. The study focused on menus served to 11–12-year-old students, selected to minimise variability in dietary needs, and because they are in the final years of primary education, when autonomy in food choices increases^(4)^. Bizkaia, a densely populated, urbanised province in northern Spain with Bilbao as its capital, features both coastal and mountainous areas. Although dietary patterns vary slightly across Spanish regions, their characteristics are similar to those of other urban areas nationwide.

### Study population and sampling

The study focused on primary schools in the Bilbao metropolitan area, where 65 288 pupils were enrolled in 2017–2018, with 81·4 % using school canteens^(17)^. Schools were selected based on (i) location within the Bilbao area, (ii) having primary pupils (ages 6–12) and (iii) provision of a catering company (on-site or external catering). These criteria ensured consistency in age group, menu access and location, facilitating comparability. The area was chosen for its high concentration of canteen users, providing a representative urban sample. Schools that declined or did not respond were excluded.

From 188 eligible primary schools, a simple random sampling method using a computer-generated number sequence was applied to minimise selection bias. A minimum of nine schools was required to achieve a ±5 % precision with 95 % CI. To ensure representativeness, the sample included equal numbers of public and charter schools.

Five of the nineteen contacted charter schools and all five contacted public schools agreed to participate, totalling ten schools (five public, five charter). These represented 3 % (3488 pupils) of Bizkaia’s primary school population, with 91 % using school canteens. Schools were mainly medium-sized (approximately 300–500 pupils in primary education). Six schools (one public, five charter) had on-site kitchens; the remaining four schools (all public) received menus prepared off-site via a hot-chain delivery system. For these schools, food transportation distances ranged from 4 to 18 km (mean: 12·2 km). Menus across all ten schools were supplied by six different catering companies. Although not identical, menus featured comparable dish types, enabling comparisons across schools.

### Data collection

Data were collected over five consecutive school days across ten different weeks during the 8-month study period (November 2017–June 2018), following prior agreements with participating schools. Special events (e.g. celebrations) were avoided to capture typical school-day menus. A total of 1000 menus were assessed (20 per d over 5 days, totalling 100 menus per school). A minimum sample size of 384 menus (95 % CI, ±5 % error, assuming maximum variability) was statistically determined and adjusted to 770 to account for clustering within schools and daily variations.

Trays were selected daily via convenience sampling by researchers and school staff. Each school’s twenty planned daily menus showed minimal differences. Only three schools (schools 8, 9 and 10) on six specific days offered students a choice for some courses (e.g. purée or soup, chicken with garlic or with tomato, ice cream or fruit). Also, when fruit was offered for dessert in these three schools, different fruit options (e.g. orange or apple) were available.

Menus included seasonal rotations due to data collection across months but maintained consistent structure and nutritional composition, ensuring minimal impact of seasonal variations.

Food intake was estimated by taking digital photographs of each student’s tray before and after intake, similar to Martinez-Perez *et al*. (2022)^(18)^. Using an iPhone 7 Plus, overhead photographs were taken at 45° (approximately 50 cm above) to capture food depth and height^(19)^. For consistency, all photographs were taken by the same researcher (R.B.-B.) under similar lighting.

Each tray was coded for accurate menu tracking. Since photographs captured only trays without identifiable student images and involved no personal data, written informed consent or ethics committee approval was not required.

In Spain, school menus constitute predefined, three-course midday meals, typically served uniformly with limited exceptions for student choice. They include a vegetable/legume/cereal-based first course, a protein-based second with a side and a fruit/dairy dessert, plus bread and water as accompaniments. Catering companies, often with dietitians, develop these menus according to national^(3)^ and regional nutritional guidelines^(20)^, which specify portion sizes, food group distribution and nutrient composition. However, decentralised implementation across regions leads to variability in application, exacerbated by structural and operational differences between public and charter schools. Public schools, under stricter government oversight, tend towards uniform procurement, while charter schools have greater flexibility in menu planning and food service contracting, impacting variety, ingredient selection and adherence to guidelines. All menu components were included in the nutritional and environmental assessments.

### Menu served, consumed and wasted assessment

Food served and consumed was estimated by visually evaluating portion sizes served and plate waste for each menu component. Portion sizes were estimated by comparing images with the photographic catalogue developed for the European Prospective Investigation into Cancer and Nutrition (EPIC) study^(21)^. Though originally designed for cancer and nutrition research, this validated manual was suitable due to its broad coverage of foods and portion sizes commonly consumed across different age groups and settings, such as Spanish school canteens. This method ensured consistency and comparability in portion estimation.

To ensure accuracy, two researchers (R.B.-B. and N.M.-P.) independently estimated portion sizes of food served and wasted. Prior to the study, both researchers underwent a training programme based on Arroyo *et al*. (2007)^(22)^, which included practical exercises with real foods to standardise visual assessment techniques and enhance inter-rater reliability across varied food types. Agreement between researchers was measured via the intraclass correlation coefficient (ICC) for each food category, with ICC > 0·85 indicating strong reliability. Most categories demonstrated strong reliability (ICC > 0·85), except vegetables in first courses (0·83) and side dishes (0·82), which were slightly lower. Bread showed perfect agreement (ICC = 1·00). Discrepancies were resolved through collaborative data review.

While most foods were covered by the photographic manual, some items (e.g. certain desserts, mixed dishes) lacked direct visual equivalents. In such cases, portion sizes were estimated using a standardised protocol: (1) referencing similar items within the manual (e.g. selecting a visually comparable portion of similar consistency or food group), (2) using standard household measures (e.g. cups, spoons), (3) consulting catering staff and (4) calculating ingredient weights from traditional recipes^(23,24)^.

Plate waste was measured using the quarter-waste visual method, an indirect 5-point visual scale (0 = 0 %, 1 = 25 %, 2 = 50 %, 3 = 75 % and 4 = 100 %) to quantify rejected food^(25)^. A full plate (100 %) indicated no consumption, while an empty plate (0 %) indicated full consumption of the food served. Waste > 25 % was considered substantial, consistent with prior research^(26)^. Non-edible parts (e.g. bones, peel, inedible skins) were excluded from estimates of both served and wasted portions at the estimation stage to analyse only edible fractions.

Quantities consumed were calculated by subtracting estimated waste from the estimated portion served for each menu component. No weighing was performed to minimise behavioural changes from monitoring. Instead, a validated photographic method was used, proven reliable in school settings where weighing is less feasible^(25)^. The same two researchers conducted all estimations to ensure consistency.

### Nutritional composition analysis

The nutritional composition of all foods served and consumed was estimated using the DIAL 2·12 food composition database for the Spanish population^(27)^. Calculated values included energy, protein, total carbohydrates (including sugars), total fat (including SFA, PUFA and MUFA), dietary fibre, Na and micronutrients (vitamins, including A, B_6_, B_12_, C, thiamine, riboflavin, niacin and folate, and minerals, including Ca, iodine, Fe, Mg, phosphorus and Zn).

To assess energy adequacy, mean daily requirements were estimated using the Institute of Medicine’s Dietary Reference Intakes, which consider Estimated Energy Requirements and activity levels^(28)^. For children aged 11–12 years with low physical activity, the recommended range is 1813–2113 kcal/d. Although physical activity was not directly measured in this study, low levels were assumed based on national^(29)^ and European data showing that only 21 % of boys and 13 % of girls in Spain meet the WHO daily activity recommendations – below European averages (27 and 21 %, respectively)^(30)^. Spain’s high childhood overweight and obesity rates further support using low activity estimates to avoid overestimation^(29)^.

School lunches are expected to provide 30 % of daily nutritional needs, based on established nutritional guidelines^(31)^. Macronutrient intake was assessed using the acceptable macronutrient distribution ranges (AMDR) for Spanish children^(3)^: 12–15 % protein, 30–35 % total fat (≤ 7 % SFA, 7–10 % PUFA and 13–18 % MUFA) and 50–60 % total carbohydrate (≤ 10 % simple carbohydrates). These recommendations align with international standards, like the WHO and FAO of the UN. Micronutrient intake was evaluated using the Institute of Medicine’s Dietary Reference Intakes^(28)^, which offer widely used benchmarks, facilitating comparability with previous international studies^(9)^.

### Carbon footprint of the food served and wasted

The CF of served and wasted food was calculated as the greenhouse gas emissions associated with food production. The CF, expressed in kilograms of CO_2_ equivalent per kilogram of menu or item (kg CO_2_-eq), was estimated using life cycle assessment methodology, aligning with ISO 14064 (2012). Among environmental indicators, CF is the most commonly used for assessing the environmental impact of dietary patterns^(16,32)^.

CF values for each food ingredient were derived from a literature review in PubMed, selecting recent and regionally relevant data. The sources and CF values for each ingredient are detailed in online supplementary material, Supplemental Table 1. Each ingredient’s CF was calculated by multiplying its quantity by its specific greenhouse gas emissions value. Total CF of a menu or food item was determined by summing the CF values of all ingredients.

The CF was assessed using a cradle-to-gate approach, encompassing all processes from raw material production to ingredient delivery to the food producer. This included (i) crops and feed production (including fertilisers, energy, fuel), (ii) animal farming, (iii) animal processing and slaughtering, (iv) packaging and (v) transporting ingredients to the food preparation facility. In accordance with ISO 14040-14044 guidelines^(33,34)^, equivalent processes for life cycle assessment comparison were excluded, such as infrastructure/machinery production, food transportation to schools, packaging and food waste disposal. Cooking, refrigeration and heating of food were also excluded due to limited data and minimal impact.

### Statistical analysis

Data analysis was performed using IBM SPSS Statistics for Windows, version 28.0 (IBM Corp.). Descriptive statistics for continuous variables were presented as means (sd) and categorical data as percentages. The normality of the distribution was assessed using the Kolmogorov–Smirnov test. In cases where the test indicated non-normality, the central limit theorem was applied, given the large sample size. This approach assumes that the sampling distribution of the mean approximates normality, even when the underlying data are not normally distributed^(35)^. Independent samples *t* tests were used to compare means between public and charter schools, while paired samples *t* tests were applied to compare the nutritional composition of served *v*. consumed menus within the same group. All tests were two-sided, with *P*-values < 0·05 considered statistically significant.

## Results

### Energy intake and nutrient composition of served and consumed menus

Table 1 presents the energy and macronutrient composition of school menus served and consumed in public and charter schools for 11- and 12-year-old students, compared with the AMDR. The energy content of menus served (791·5 (sd 176·7) kcal) exceeded the AMDR, while the energy consumed (598·4 (sd 203·7) kcal) was significantly lower due to food waste (*P* < 0·001). Despite this reduction, consumed energy remained above the recommendations. On average, served menus provided 40·6 % of the total energy, while consumed menus accounted for 30·7 %, as shown in Table 2.

**Table 1.** Energy and macronutrient composition of school menus served (*n* 1000 menus) and consumed (*n* 1000 menus) in school canteens among 11–12-year-old children from public (*n* 500 menus) and charter (*n* 500 menus) primary schools

	Guidelines (kilocalories or grams)*	Menus served							Menus consumed							*P*-value^**‡**^
		Total (*n* 1000)		Public (*n* 500)		Charter (*n* 500)		*P*-value^†^	Total (*n* 1000)		Public (*n* 500)		Charter (*n* 500)		*P*-value^†^	
		Mean	sd	Mean	sd	Mean	sd		Mean	sd	Mean	sd	Mean	sd		
Energy (kcal)	543·9–633·9^§^	791·5	176·7	754·9	162·3	828·2	183·3	**< 0·001**	598·4	203·7	553·0	193·2	643·9	203·9	**< 0·001**	**< 0·001**
Total fat (g)	17·5–23·8	39·7	13·4	37·6	12·8	41·8	13·7	**< 0·001**	30·7	13·9	27·7	13·2	33·7	13·9	**< 0·001**	**< 0·001**
SFA (g)	≤ 4·8	9·1	4·8	8·8	4·8	9·5	4·7	**0·017**	7·3	4·5	6·8	4·4	7·9	4·5	**< 0·001**	**< 0·001**
MUFA (g)	7·6–12·2	24·0	12·5	21·3	6·6	26·8	15·9	**< 0·001**	18·5	12·2	15·3	7·0	21·6	15·2	**< 0·001**	**< 0·001**
PUFA (g)	4·1–6·8	4·9	2·0	4·3	1·8	5·4	2·0	**< 0·001**	3·8	2·0	3·2	1·8	4·4	2·0	**< 0·001**	**< 0·001**
Proteins (g)	15·7–23·0	29·7	10·0	29·4	10·4	30·0	9·6	0·377	23·1	10·2	22·7	11·1	23·4	9·3	0·317	**< 0·001**
Total carbohydrates (g)	65·6–91·9	74·7	18·1	70·4	16·4	79·0	18·8	**< 0·001**	54·6	20·5	50·2	19·3	59·1	20·8	**< 0·001**	**< 0·001**
Simple carbohydrates (g)	≤ 15·3	18·9	5·9	18·7	6·7	19·0	5·1	0·522	14·3	7·3	13·8	7·7	14·7	6·9	0·054	**< 0·001**

Note: ^*^Institute of Medicine (2006), FAO of the UN (2019) and Sociedad Española de Nutrición Comunitaria (2008). ^†^Student’s *t* test was used to compare means between the two types of schools. Significant *P*-values are highlighted in bold. ^‡^Paired *t* test was used to compare mean values between served and consumed menus, in order to quantify the nutritional gap between what is offered and what is actually consumed by students. Significant *P*-values are highlighted in bold. ^§^As a reference, the mean of the Estimated Energy Requirement of 2113 and 1813 kcal/d was used.

**Table 2.** Energy contribution of macronutrients in school menus (*n* 1000 menus) and consumed (*n* 1000 menus) by 11–12-year-old children, compared with nutritional guidelines (% of total energy intake), from public (*n* 500 menus) and charter (*n* 500 menus) primary schools

	Guidelines (% energy)*	Menus served						Menus consumed					
		Total (*n* 1000)		Public (*n* 500)		Charter (*n* 500)		Total (*n* 1000)		Public (*n* 500)		Charter (*n* 500)	
		Mean	sd	Mean	sd	Mean	sd	Mean	sd	Mean	sd	Mean	sd
Energy	30 % of total daily energy	40·6	9·1	38·7	8·3	42·4	9·4	30·7	10·4	28·4	9·9	33·0	10·4
Total fat	30–35 %	44·4	6·7	44·0	6·6	44·8	6·8	44·9	9·5	43·6	10·2	46·3	8·6
SFA	≤ 7 %	10·0	3·7	9·9	3·7	10·1	3·8	4·7	1·9	10·8	2·5	13·7	11·6
MUFA	13–18 %	27·4	15·4	25·1	3·3	29·6	21·3	12·2	8·5	10·8	2·5	13·7	11·6
PUFA	7–10 %	5·6	2·1	5·2	2·1	6·0	2·1	2·5	1·2	2·3	1·2	2·8	1·1
Proteins	12–15 %	15·1	4·0	15·5	3·8	14·6	4·1	15·4	4·7	16·2	4·7	14·7	4·6
Total carbohydrates	50–60 %	38·4	7·5	38·1	7·9	38·6	7·1	37·6	10·5	38·0	12·0	37·2	8·8
Simple carbohydrates	≤ 10 %	10·0	3·7	10·3	4·1	9·6	3·3	10·5	6·8	11·2	8·0	9·8	5·4

Note: *Institute of Medicine (2006), FAO of the UN (2019) and Sociedad Española de Nutrición Comunitaria (2008).

Fats exceeded the AMDR in both served (39·7 (sd 13·4) g, 44·4 % of energy) and consumed (30·7 (sd 13·9) g, 44·9 % of energy) menus, particularly for SFA (9·1 (sd 4·8) g served, 7·3 (sd 4·5) g consumed) and MUFA (24·0 (sd 12·5) g served, 18·5 (sd 12·2) g consumed). Protein also exceeded recommendations in both served (29·7 (sd 10·0) g 15·1 % of energy) and consumed menus (23·1 (sd 10·2) g, 15·4 % of energy), although food waste reduced it closer to adequacy. Total carbohydrates consumed (54·6 (sd 20·5) g, 37·6 % of energy) frequently fell below the AMDR, while simple sugars (14·3 (sd 7·3) g) generally met recommendations.

Comparisons between public and charter schools showed significant nutritional differences. Charter schools served and consumed menus with significantly higher energy, total fat, SF and carbohydrate content (all *P* < 0·001). No significant differences were observed for protein and simple sugars intake (*P* > 0·05).

A broader analysis of macronutrient adequacy (Table 3) showed that 79·2 % of served and 40·0 % of consumed menus exceeded recommended energy levels. Similarly, fats exceeded the AMDR in 93·7 % of served and 66·8 % of consumed menus, with similar trends observed for SFA and MUFA. PUFA were under-consumed in 65·6 % of menus. About one-third of menus met protein recommendations (32·6 % served, 32·7 % of consumed), while most exceeded them (66·0 % served, 43·0 % consumed). Carbohydrate adequacy was low, with only 22·6 % of consumed menus meeting recommendations and 72·4 % falling below recommendations.

**Table 3.** Percentage of school menus served (*n* 1000) and consumed (*n* 1000) meeting, falling below or exceeding energy and macronutrient adequacy to acceptable macronutrient distribution ranges among 11–12-year-old children

	Guidelines*	Menus served (*n* 1000)			Menus consumed (*n* 1000)		
		% adequacy	% below adequacy^†^	% over adequacy	% adequacy	% below adequacy^†^	% over adequacy
Energy	543·9–633·9 kcal^‡^	15·4	5·4	79·2	17·2	42·8	40·0
Total fat	17·5–23·8 g	5·3	1·0	93·7	17·0	16·2	66·8
SFA	≤ 4·8 g	19·4	NA	80·6	33·2	NA	66·8
MUFA	7·6–12·2 g	2·1	0·0	97·9	17·6	7·7	74·7
PUFA	4·1–6·8 g	47·2	39·9	12·9	26·7	65·6	7·7
Proteins	15·7–23·0 g	32·6	1·4	66·0	32·7	24·3	43·0
Total carbohydrates	65·6–91·9 g	37·0	39·6	23·4	22·6	72·4	5·0
Simple carbohydrates	≤ 15·3 g	28·9	NA	71·1	50·0	NA	50·0

Note: *Institute of Medicine (2006), FAO of the UN (2019) and Sociedad Española de Nutrición Comunitaria (2008). ^†^Not applicable (NA) due to the absence of a lower threshold for the nutrient. ^‡^As a reference, the mean of the Estimated Energy Requirement of 2113 and 1813 kcal/d was used.

Table 4 presents the composition and adequacy of dietary fibre, Na, vitamins and minerals in served and consumed menus in public and charter schools. Niacin and vitamin B_6_ were adequately served (100 % adequacy) and consumed (> 80 % adequacy). In contrast, Ca and iodine showed major deficiencies, with only 1·1 % of menus served and consumed meeting Ca recommendations. Dietary fibre adequacy was also low (42·1 % served, 21·7 % consumed; *P* < 0·001). Mg and Fe adequacy approached recommended levels in served menus (90·8 and 95·9 %, respectively) but dropped significantly in consumed menus (*P* < 0·001), especially Mg (44·9 %; *P* < 0·001). Na exceeded recommendations in both served (980·4 (sd 302·2) mg) and consumed (717·9 (sd 306·4) mg) (*P* < 0·001).

**Table 4.** Composition and adequacy of dietary fibre, sodium, vitamins and minerals of menus served (*n* 1000) and consumed (*n* 1000) among 11–12-year-old children from public (*n* 500) and charter (*n* 500) primary schools

	Guidelines*	Menus served								Menus consumed								*P*-value^**‡**^
		Total (*n* 1000)		Public (*n* 500)		Charter (*n* 500)		*P*-value^†^	% adequacy	Total (*n* 1000)		Public (*n* 500)		Charter (*n* 500)		*P*-value^†^	% adequacy	
		Mean	sd	Mean	sd	Mean	sd		Total	Mean	sd	Mean	sd	Mean	sd		Total	
Dietary fibre (g)	8·55	8·80	3·65	9·09	3·55	8·50	3·74	**0·011**	42·1	6·02	3·49	6·15	3·79	5·90	3·16	0·247	21·7	**< 0·001**
Na (mg)	<540·00	980·43	302·26	891·79	212·07	1069·07	349·54	**<0·001**	0·0	717·92	306·43	619·56	242·14	806·27	337·20	**<0·001**	0·0	**<0·001**
Vitamin A (µg)	180·00	228·95	143·81	247·63	158·00	210·28	125·47	**<0·001**	52·6	128·14	105·44	124·48	121·64	131·79	86·22	0·247	20·5	**<0·001**
Thiamine (mg)	0·27	0·44	0·23	0·44	0·25	0·44	0·20	0·990	80·3	0·33	0·21	0·32	0·22	0·34	0·18	0·205	52·8	**<0·001**
Riboflavin (mg)	0·27	0·45	0·16	0·46	0·16	0·45	0·16	0·395	86·9	0·35	0·16	0·35	0·16	0·35	0·16	0·770	67·0	**<0·001**
Niacin (mg)	3·60	12·89	3·74	12·57	3·59	13·20	3·87	**0·008**	100·0	9·91	4·10	9·48	4·08	10·33	4·08	**0·001**	94·6	**<0·001**
Vitamin B_6_ (mg)	0·30	0·67	0·21	0·70	0·17	0·63	0·25	**<0·001**	100·0	0·49	0·21	0·51	0·21	0·48	0·21	**0·016**	83·6	**<0·001**
Folate (µg)	90·00	106·89	49·40	117·27	53·17	96·50	42·91	**<0·001**	60·0	73·45	45·85	78·59	54·42	68·31	34·55	**<0·001**	27·7	**<0·001**
Vitamin B_12_ (µg)	0·54	2·96	3·83	3·09	3·74	2·83	3·92	0·272	81·9	2·44	3·31	2·45	3·02	2·44	3·57	0·984	75·8	**<0·001**
Vitamin C (mg)	13·50	37·76	25·74	42·63	25·88	32·88	24·68	**<0·001**	81·8	20·66	16·93	23·40	17·98	17·91	15·34	**<0·001**	57·2	**<0·001**
Ca (mg)	390·00	175·53	96·97	166·52	75·68	184·55	113·73	**0·003**	1·1	136·79	90·74	125·48	74·33	148·10	103·46	**<0·001**	1·1	**<0·001**
Iodine (µg)	36·00	38·32	41·93	32·02	20·30	44·63	55·02	**<0·001**	28·0	28·01	31·69	22·47	16·44	33·55	40·97	**<0·001**	15·8	**<0·001**
Fe (mg)	2·40	4·92	1·60	5·02	1·55	4·81	1·65	**0·037**	95·9	3·58	1·62	3·60	1·77	3·56	1·46	0·667	73·7	**<0·001**
Mg (mg)	72·00	94·90	19·70	95·51	16·70	94·29	22·30	0·330	90·8	69·42	23·38	67·83	25·60	71·01	20·84	**0·031**	44·9	**<0·001**
Phosphorus (mg)	375·00	441·86	135·03	428·07	91·43	455·64	166·63	**0·001**	71·1	344·99	134·66	329·71	117·59	360·28	148·35	**<0·001**	39·2	**<0·001**
Zn (mg)	2·40	3·26	1·97	3·61	2·40	2·92	1·33	**<0·001**	54·8	2·57	1·88	2·82	2·28	2·32	1·32	**<0·001**	34·6	**<0·001**

Note: *Dietary reference intakes (1997) for phosphorus and Mg; dietary reference intakes (1998) for thiamine, riboflavin, niacin, vitamin B_6_, folate and vitamin B_12_; dietary reference intakes (2000) for vitamin C and vitamin E; dietary reference intakes (2001) for vitamin A, iodine, Fe and Zn; dietary reference intakes (2005) for fibre; dietary reference intakes (2011) for Ca; and dietary reference intakes (2019) for Na. ^†^Student’s *t* test was used to compare means between the two types of schools. Significant *P*-values are highlighted in bold. ^‡^Paired *t* test was used to compare means between served and consumed menus, in order to quantify the nutritional gap between what is offered and what is actually consumed by students. Significant *P*-values are highlighted in bold.

Differences in nutrient provision and intake were also noted between school types. Public schools provided higher levels of dietary fibre, vitamin A, B_6_, C, folate, Fe and Zn than charter schools (*P* < 0·001), although some differences diminished when consumed. Charter schools showed better provision and intake of niacin, Ca, iodine and phosphorus (*P* < 0·001) than public schools. Na levels were significantly higher in both served and consumed menus in charter schools (*P* < 0·001).

### Food served and wasted

Table 5 shows the mean amounts of food served and wasted by food category and school type. On average, 523·9 g of edible food was served per menu (±71·6 g), with public schools serving slightly more (532·4 (sd 66·9) g) than charter schools (515·5 (sd 75·1) g). The most frequently served first courses were starchy foods (e.g. pasta with tomato, paella), while second courses often featured meat (e.g. roasted chicken, burgers), typically accompanied by vegetables such as salad or roasted peppers. Desserts were mainly fresh fruit – especially apples and oranges – followed by sugar-sweetened yoghurts.

**Table 5.** Mean amounts (in grams) of food served and wasted and percentage of menus with > 25 % waste in school lunches, by food category and school type (public and charter)

	n*	Served						Wasted									
		Total		Public		Charter		Total			Public			Charter			*P*-value^§^
		Edible mean weight^†^, g	sd	Edible mean weight^†^, g	sd	Edible mean weight^†^, g	sd	Edible mean weight^†^, g	sd	% with > 25 % waste^‡^	Edible mean weight^†^, g	sd	% with > 25 % waste^‡^	Edible mean weight^†^, g	sd	> % with > 25 % waste^‡^	
Total	1000	523·9	71·6	532·4	66·9	515·5	75·1	140·5	100·7	12·2	151·5	112·3	16·6	129·5	86·3	7·8	**< 0·001**
First course																	
Legumes	300	177·5	22·8	176·2	27·4	178·9	15·9	48·4	55·4	26·0	46·5	63·0	28·1	50·6	45·3	23·6	0·222
Starchy foods (i.e. rice, pasta, potatoes)	426	218·2	45·5	219·3	53·1	217·3	39·1	42·2	65·3	14·3	53·8	77·1	19·4	33·6	53·6	10·6	**0·008**
Vegetables	274	213·5	48·2	237·6	47·0	179·6	23·0	71·3	78·9	38·0	74·4	89·9	37·5	66·8	60·2	38·6	0·476
Second course																	
Eggs	120	156·2	28·6	176·0	3·0	116·5	7·6	34·0	50·9	22·5	43·4	58·4	27·5	15·2	21·0	12·5	**0·049**
Fish	300	144·2	44·6	111·3	27·2	172·9	36·3	35·4	47·1	26·3	30·9	42·1	30·0	39·4	50·8	23·1	0·112
Meat	420	137·4	42·1	144·2	45·4	128·3	35·4	22·2	37·4	18·3	23·9	41·5	18·3	19·9	30·9	18·3	0·522
Starchy foods (i.e. croquettes, Spanish pie ‘empanadilla’)	160	157·6	28·6	127·5	12·7	167·7	25·2	10·9	26·1	8·1	3·6	13·3	5·0	13·4	28·8	9·2	0·323
Side dish																	
Legumes	20	25·5	1·3	25·5	25·2	–		24·9	3·2	100·0	24·9	3·2	100·0	–		–	–
Starchy foods	80	39·5	15·0	43·8	0·8	35·1	20·4	12·4	14·5	27·5	18·5	15·6	40·0	6·2	10·2	15·0	**0·011**
Vegetables	660	31·5	9·9	30·4	8·7	33·1	11·2	25·2	14·1	82·7	23·9	13·3	81·5	27·1	15·0	84·6	0·177
Dessert																	
Dairy products	289	125·0	0·0	125·0	0·0	125·0	0·0	1·7	12·4	1·0	2·5	14·9	1·3	0·7	8·2	0·8	0·580
Fruits	614	104·7	36·8	106·9	30·6	102·4	42·6	43·9	43·5	55·4	43·5	41·6	53·1	44·5	45·6	57·8	0·138
Other desserts (i.e. doughnut, custard)	97	97·5	28·6	125·0	0·0	90·4	28·0	14·4	35·8	12·4	53·1	53·7	40·0	4·3	20·0	5·2	**< 0·001**
Accompaniment																	
Starchy food: bread	1000	40·0	0·0	40·0	0·0	40·0	0·0	15·7	16·9	43·2	13·7	14·8	39·2	17·8	18·5	47·2	**0·006**

Note: *The *n* values represent the total number of menus assessed (*n* 1000) and the number of menus in which each specific food category was served. For example, *n* 300 for legumes indicates that legumes were served as a first course in 300 of the 1000 menus observed. ^‡^Edible mean weight refers to the mean weight of the edible portion of food items, excluding inedible components such as bones, peel or shells. This measure was used to ensure that both nutritional analysis and food waste estimates reflect only the portion of food intended for consumption. The inedible parts were excluded during visual estimation. ^**†**^Percentages of menus/food items with > 25 % waste are shown. ^**§**^Student’s *t* test was used to compare the means of > 25 % wasted between the two types of schools. Significant *P*-values are highlighted in bold.

Food waste averaged 140·5 g per menu (±100·7 g), with 12·2 % of menus showing > 25 % waste. Public schools had significantly higher food waste (151·5 (sd 112·3) g) than charter schools (129·5 (sd 86·6) g; *P* < 0·001) and more menus with > 25 % waste (16·6 % *v*. 7·8 %; *P* < 0·001). When analysing specific food categories, legumes and vegetables showed notable waste, particularly as side dishes (> 80 % waste in both school types), and around one-third as first courses. Second-course items, like eggs, fish and meat, also presented substantial waste, with fish showing the highest waste (26·3 %). Among desserts, fruits had the most waste (55·4 %), with no significant differences between school types (*P* > 0·05). Bread as an accompaniment also showed high waste (43·2 %), with charter schools wasting more (47·2 % *v*. 39·2 %; *P* < 0·001). Further examining specific food items, starchy foods (e.g. pasta, croquettes, empanadillas) and sugar-sweetened yoghurts were among the least wasted. In contrast, the highest waste was observed for vegetables – particularly when served whole rather than puréed – grilled fish, peas (as a side dish) and fruits, particularly oranges and pears.

Online supplementary material, Supplemental Table 2 presents mean nutrient losses due to food waste and the corresponding percentage reduction from served to consumed menus. Energy dropped by 24·4 %, with similar reductions in fats (–22·7 %), proteins (–22·2 %) and total carbohydrates (–26·9 %). Dietary fibre showed a notable decrease (–31·5 %), as did vitamins, like vitamin A (–44·0 %) and C (45·3 %), and minerals, like Fe (27·2 %) and iodine (26·9 %).

### Carbon footprint of food served and wasted

Table 6 presents the CF (kg CO_2_-eq/total menu or plate) of food served and wasted, by food category and school type. The mean CF of the served menu was 1·489 (sd 1·210) kg CO_2_-eq, while that of the wasted food was 0·298 (sd 0·476) kg CO_2_-eq. Protein-rich second-course items contributed most, especially meat (1·717 (sd 1·61) kg CO_2_-eq/plate served; 0·263 (sd 0·434) kg CO_2_-eq/plate wasted) and fish (1·058 (sd 0·581) kg CO_2_-eq/ plate served; 0·250 (sd 0·581) kg CO_2_-eq/plate wasted). Among first courses and side dishes, starchy foods (e.g. rice, pasta) had the highest served emissions, while vegetables and legumes had higher wasted emissions due to greater waste.

**Table 6.** Carbon footprint (kg CO_2_-eq per menu or plate) of total menus and food items (grouped by food category) served and wasted in school lunches, by school type (public and charter)

	*n* ^*,‡^	Served							Wasted						
		Total		Public		Charter		*P*-value^†^	Total		Public		Charter		*P*-value^†^
		Mean	sd	Mean	sd	Mean	sd		Mean	sd	Mean	sd	Mean	sd	
Total	1000	1·489	1·210	1·597	1·495	1·381	0·820	**0·005**	0·298	0·476	0·312	0·542	0·285	0·398	0·374
First course															
Legumes	300	0·132	0·098	0·073	0·056	0·200	0·092	**< 0·001**	0·033	0·043	0·019	0·037	0·049	0·043	**< 0·001**
Starchy foods (i.e. rice, pasta, potatoes)	426	0·234	0·291	0·219	0·362	0·246	0·226	0·351	0·052	0·149	0·057	0·183	0·048	0·118	0·542
Vegetables	274	0·132	0·165	0·122	0·152	0·146	0·182	0·237	0·073	0·143	0·061	0·117	0·091	0·173	0·118
Second course															
Eggs	120	0·227	0·096	0·194	0·003	0·292	0·146	**< 0·001**	0·045	0·062	0·048	0·064	0·039	0·060	0·477
Fish	300	1·058	0·581	0·709	0·217	1·364	0·627	**< 0·001**	0·263	0·434	0·193	0·275	0·325	0·528	**0·003**
Meat	420	1·717	1·61	2·106	1·874	1·198	0·965	**< 0·001**	0·250	0·581	0·310	0·714	0·171	0·313	**0·007**
Starchy foods (i.e. croquettes, Spanish pie ‘empanadilla’)	160	0·417	0·161	0·416	0·044	0·418	0·185	0·901	0·031	0·077	0·012	0·043	0·037	0·085	**0·016**
Side dish															
Legumes	20	0·018	0·000	0·018	0·000	–		–	0·018	0·002	0·018	0·002	–		–
Starchy foods	80	0·060	0·071	0·018	0·003	0·103	0·081	**< 0·001**	0·014	0·025	0·007	0·006	0·020	0·339	**0·013**
Vegetables	660	0·020	0·006	0·019	0·006	0·021	0·006	**< 0·001**	0·016	0·009	0·015	0·009	0·018	0·009	**< 0·001**
Dessert															
Dairy products	289	0·179	0·000	0·179	0·000	0·179	0·000	1·000	0·002	0·018	0·004	0·021	0·001	0·011	0·191
Fruits	614	0·049	0·027	0·048	0·024	0·051	0·030	0·280	0·018	0·019	0·019	0·020	0·016	0·018	0·079
Other desserts (i.e. doughnut, custard)	97	0·237	0·101	0·350	0·000	0·198	0·089	**< 0·001**	0·036	0·094	0·149	0·150	0·007	0·038	**< 0·001**
Accompaniment															
Starchy food: bread	1000	0·062	0·000	0·062	0·000	0·062	0·000	1·000	0·024	0·026	0·021	0·023	0·027	0·029	**< 0·001**

Note: *The *n* values represent the total number of menus assessed (*n* 1000) and the number of menus in which each specific food category was served. For example, *n* 300 for legumes indicates that legumes were served as the first course in 300 of the 1000 menus observed. ^‡^Carbon footprint values represent the total emissions of the full dish, calculated from all ingredients used, not just the main food group. Emission factors of all ingredient use are detailed in online supplementary material, Supplemental Table 1. ^†^Student’s *t* test was used to compare means between the two types of schools. Significant *P*-values are highlighted in bold.

Public schools had a significantly higher mean CF for served menus (1·597 (sd 1·495) kg CO_2_-eq) than charter schools (1·381 (sd 0·820) kg CO_2_-eq) (*P* < 0·05), with no significant differences in wasted emissions (*P* > 0·05). Charter schools had higher served emissions for first courses and side dishes (*P* < 0·05), except for starchy foods and vegetables as a first course and legumes as a side dish (*P* > 0·05). Public schools had higher served emissions for meat, while charter schools had higher served emissions for fish (*P* < 0·05).

## Discussion

This study assessed the nutritional composition, adequacy and environmental impact of menus served, consumed and wasted by 11–12-year-old students in public and charter schools in northern Spain. Menus were high in energy, fats, proteins and Na but low in carbohydrates, fibre and certain micronutrients. Food waste, though reducing excess intake, caused nutrient losses, especially from discarded fruits and vegetables. The CF averaged 1·489 kg CO_2_-eq, mainly from meat and fish. Public schools served more nutrient-dense menus but showed higher food waste than charter schools.

Menus supplied 40·6 % of daily energy needs instead of the recommended 30 %, aligning with similar studies in other regions of Spain^(36)^ and international studies, including from the USA and South Korea^(37,38)^. Only 15·4 % of menus served met energy AMDR, in line with Liz Martins *et al*. (2021)^(9)^, who reported just 12·5 % compliance in Portuguese schools. However, while their study attributed energy insufficiency to a lack of carbohydrate-rich foods, the main issue in the present study was excessive energy from fat-rich foods, as shown by the macronutrient composition.

Food waste mitigated over-nutrition by reducing consumption to 30·7 % of daily needs. This aligns with findings from Portugal^(39)^ and Sweden^(40)^, where school menus provided around 27 % of daily energy. Higher energy values in Spain may reflect cultural dietary patterns, such as the frequent use of calorie-dense foods like olive oil in the Mediterranean diet and increased meat consumption beyond traditional recommendations^(40)^. However, while waste reduced excess intake, it also compromised nutritional adequacy and environmental sustainability.

The macronutrient composition showed significant imbalances, with fat, particularly SFA and MUFA, exceeding AMDR in both served and consumed menus. This likely reflects a menu rich in energy-dense, fat-rich foods – like fried items and meats – while underrepresenting fibre-rich carbohydrate sources like whole grains and legumes. Similar trends were noted internationally^(41)^. High MUFA in this study likely reflects the common use of olive oil in Spanish cuisine, while low PUFA may stem from limited fish inclusion in school menus^(42)^.

Protein exceeded recommendations in both served (66 %) and consumed (43 %) menus. Although food waste slightly reduced intake, over-provision persisted – likely due to high use of animal-based proteins^(41)^, as seen in other international studies^(9,41)^.

In contrast, carbohydrate was frequently insufficient, with consumed menus providing only 37·6 % of energy from carbohydrates and 72·4 % falling below recommendations, consistent with other studies^(9,39,41,43)^. While simple sugars often met recommendations, fibre-rich complex carbohydrates were lacking, likely due to limited whole grains and frequent vegetable waste and a preference for protein- and fat-rich foods. High vegetable waste likely contributed to the significant decline in dietary fibre adequacy, from 42·1 % in served to 21·7 % in consumed menus, highlighting the challenge of meeting fibre intake requirements in school menus, as observed in studies from Portugal^(9)^ and Sweden^(8)^.

Micronutrient gaps were notable, especially for Ca and iodine. Although menus alone did not provide sufficient Ca, similar to previous studies^(8,9,36)^, this was not considered a major concern, as dairy product consumption outside school, particularly at breakfast in many European countries, often compensates^(43)^. Despite Ca’s lower contribution from lunch, it was included in the analysis for a complete micronutrient profile. Regarding iodine, while previous studies^(9,36)^ reported low adequacy, values in this study were closer to recommendations. This is likely due to Spain’s widespread use of iodised salt^(44)^. However, as iodised salt use was not systematically recorded, iodine intake might be over- or under-estimated. Continued monitoring remains important despite the absence of a critical deficiency.

Some nutrients, like niacin and vitamin B_6_, met adequacy targets, suggesting they were well-integrated into menus and accepted by students. Conversely, while Mg was adequately provided, its consumption adequacy significantly dropped, consistent with Osowski *et al*. (2015)^(8)^. This reduction is likely due to low acceptance and high waste of magnesium-rich vegetables, such as leafy greens.

In contrast, Na levels in both served and consumed menus exceeded recommendations, despite a notable drop in intake. This aligns with other studies, where Na intake often surpasses recommendations due to the extensive use of processed foods^(9,43)^, like croquettes, empanadillas and crisps.

Despite shared government guidelines, significant nutritional differences were observed between public and charter schools. The non-binding nature of these guidelines, combined with varying enforcement, catering practices and oversight, led to inconsistent compliance. Public schools, operating under stricter regulations and tighter budgets, offered more nutrient-dense, cost-effective menus, resulting in higher fibre and micronutrient levels but also lower student appeal and greater waste. In contrast, charter schools, with more autonomy and funding, often prioritised palatable, energy-dense options. This improved intake of nutrients like Ca and phosphorus but also led to excessive energy, macronutrient and Na levels, often from processed foods (e.g. fried croquettes, custard, doughnuts). These patterns align with previous national^(45)^ and international studies^(46)^.

Food waste results highlighted significant nutritional and environmental implications. The mean serving size was 523·9 g/meal, with public schools providing slightly more than charter schools. As Biasini *et al*. (2024) noted, despite menus being designed to meet guidelines, variability in served portions can occur due to factors like students requesting modifications^(18)^ and lack of standardised serving protocols^(47)^. Food waste averaged 140·5 g/meal, with 12·2 % of menus exceeding 25 % waste, aligning with findings from Italian primary schools (mean 138·6 g^(16)^ and 136 g^(48)^). Energy loss reached 24·4 % in this study, consistent with previous findings reporting a 26 % loss^(9,16)^.

Vegetables and legumes (especially as side dishes), followed by fruits, had the highest waste, with over half of menus exceeding 25 % waste. This resulted in notable losses in macronutrients (from –22·2 to –26·9 %), dietary fibre (–31·5 %), vitamins like vitamin A (–44·0 %) and vitamin C (–45·3 %) and minerals like Fe (–27·2 %) and iodine (–26·9 %). These losses emphasise the need to address food waste to ensure students receive the intended nutritional benefits. High waste of vegetables and fruit has been widely reported^(10,16)^. Strategies like improved menu planning, portion control and adapting presentation of less-preferred foods could reduce waste and improve nutritional adequacy.

Similar to García-Herrero *et al*. (2019)^(48)^, who reported a 1·50 kg CO_2_-eq footprint for primary schools’ menus, this study’s served menus averaged 1·489 kg CO_2_-eq, with meat and fish contributing the highest emissions. This aligns with other studies that identified animal-based foods as the most carbon-intensive in school canteens^(32)^. The CF of wasted menus was 0·298 kg CO_2_-eq, similar to the 0·2–0·3 kg CO_2_-eq range reported by Biasini *et al*. (2024)^(16)^.

In terms of school type, public schools had a significantly higher CF for served menus than charter schools (1·597 *v*. 1·381 kg CO_2_-eq, respectively), mainly due to larger portions and food composition. Despite greater food waste in public schools, emissions did not differ significantly between school types, likely due to differences in waste composition. Reducing high-emission food waste could mitigate the environmental impact of menus across both types of schools. Additionally, reliance on off-site kitchens in public schools likely increased transport-related emissions, highlighting the sustainability benefits of on-site preparation in charter schools.

The results underscore the challenge of balancing nutritional guidelines with student preferences in school menus. While guidelines aim to ensure adequate nutrient intake, food is not always served in correct portions or is rejected due to taste, leading to waste, lower consumption and greater environmental impact. As observed in this study, fibre- and micronutrient-rich menus may meet standards but are poorly received, whereas palatable, energy-dense options often exceed limits for calories, fats and Na. Ensuring menus are nutritious, appealing and sustainable remains a challenge. Schools may need strategies combining curriculum-integrated nutrition education (focused on food waste), improved portion control and flexible meal planning. Offering diverse, lower-emission and student-preferred options that meet dietary standards, along with involving students in meal preparation and decisions, could improve acceptance, enhance nutritional compliance and reduce emissions^(49)^.

To contextualise these findings within the European framework, nutrient intakes were compared with the Dietary Reference Values established by the European Food Safety Authority^(50)^. The European Food Safety Authority provides macronutrient reference ranges (e.g. 45–60 % energy from carbohydrates, 20–35 % from fats, 10–15 % from protein for 11–14 years), which align closely with the AMDR used in this study. Similarly, the European Food Safety Authority’s micronutrient Population Reference Intakes like Ca (1150 mg/d), Fe (11 mg/d) and vitamin C (70 mg/d) are broadly comparable to Institute of Medicine values^(32)^, though with slight differences due to regional and methodological factors. These discrepancies reflect methodological and regional dietary assumptions. Despite these variations, both systems highlight similar nutritional priorities. The consistent inadequacies in fibre, Ca and certain vitamins reinforce the robustness of this study’s findings and underscore the need for improved school meal planning.

Several limitations should be considered when interpreting these results. First, the study’s geographical scope in northern Spain may limit the applicability to other regions with different dietary habits, food systems or school meal programmes. Second, while data were collected between November 2017 and June 2018, findings remain relevant. Limited national policy changes, fragmented regional guidance and minimal operational shifts in the studied schools suggest consistent trends. Furthermore, recent research in Spain^(11,45)^ and Europe^(16)^ confirms the persistence of the challenges identified (e.g. excess calories, insufficient fibre and high vegetable waste), supporting the continued relevance of these findings. Third, reliance on visual estimation introduces potential bias in quantities, especially for complex dishes not in the photographic manual, despite standardised procedures. Additionally, while the quarter-waste method is widely used, it may slightly overestimate plate waste, especially when food is dispersed or mixed on the plate. Fourth, salt quantity estimation from standard recipes and catering staff input, without systematic recording of actual iodised salt use, may have led to under- or overestimation of Na and iodine. Finally, the environmental impact assessment was limited to the CF of food items, excluding energy used for preparation, storage and transportation, potentially underestimating the total footprint.

Despite its limitations, this study offers several strengths. It provides a comprehensive view of school lunches by analysing food served, consumed and wasted, linking nutritional quality with environmental impact. The comparison between public and charter schools reveals how institutional factors shape outcomes like nutrient adequacy, food waste and emissions. The use of standardised visual estimation techniques, combined with rigorous training and reliability checks, enhances data credibility. Finally, the methodological framework employed is adaptable to other settings, increasing the study’s transferability and contributing to the global debate on nutrition and sustainability.

### Conclusion

This study revealed key issues in the nutritional quality and environmental impact of school menus in primary public and charter schools. Menus often exceeded energy and fat recommendations while lacking fibre and several micronutrients. Food waste further reduced nutritional value and increased environmental burden. Differences between school types highlight the need for targeted strategies to improve both dietary balance and sustainability.

## Supporting information

Martinez-Perez et al. supplementary material Martinez-Perez et al. supplementary material

## Data Availability

Data will be made available on request.

## Supplementary material

For supplementary material accompanying this paper, visit https://doi.org/10.1017/S1368980025101158.
