# Supplementary material for: Nutritional and environmental assessment of school menus served, consumed and wasted in primary schools in Spain: a comparison of public and charter schools
Source: Public Health Nutr. 2025 Sep 19;28(1):e172. doi: 10.1017/S1368980025101158 (PMC12722078; doi:10.1017/S1368980025101158)
Supplement: Martinez-Perez et al. supplementary material [file S1368980025101158sup001.docx]

[**Supplementary material**](https://www.sciencedirect.com/science/article/pii/S0048969720324566?via%3Dihub#s0075)

**Supplementary Table 1.** Carbon footprint of all ingredients used and the source from which it was obtained.

| **Food group** | **Food type** | **CF factor**  **(kg CO2/kg food type)** | **Source^*^** |
| --- | --- | --- | --- |
| Vegetables |  |  |  |
|  | Asparagus | 0.92 | Clune et al., 2017 |
|  | Beans | 0.30 | Clune et al., 2017 |
|  | Carrot | 0.22 | Clune et al., 2017 |
|  | Carrot | 0.22 | Clune et al., 2017 |
|  | Cauliflower | 0.35 | Clune et al., 2017 |
|  | Garlic | 0.57 | Clune et al., 2017 |
|  | Green beans | 0.51 | Clune et al., 2017 |
|  | Leek | 0.18 | Clune et al., 2017 |
|  | Lettuce | 0.38 | Clune et al., 2017 |
|  | Mushrooms | 0.27 | Clune et al., 2017 |
|  | Olives | 0.6 | Clune et al., 2017 |
|  | Onion | 0.18 | Clune et al., 2017 |
|  | Pepper | 0.6 | Clune et al., 2017 |
|  | Pumpkin | 0.33 | Clune et al., 2017 |
|  | Spinach | 0.54 | Clune et al., 2017 (from Swiss chard) |
|  | Swiss chard | 0.54 | Clune et al., 2017 |
|  | Tomato | 0.46 | Clune et al., 2017 |
|  | Zucchini | 0.42 | Clune et al., 2017 |
| Legumes |  |  |  |
|  | Beans | 0.62 | Clune et al., 2017 |
|  | Lentils | 1.03 | Clune et al., 2017 |
|  | Peas | 0.6 | Clune et al., 2017 |
| Egg |  |  |  |
|  | Eggs | 3.39 | Clune et al., 2017 |
| Fruit |  |  |  |
|  | Apple | 0.36 | Clune et al., 2017 |
|  | Banana | 0.79 | Clune et al., 2017 |
|  | Kiwi | 0.47 | Clune et al., 2017 |
|  | Mandarin | 0.45 | Clune et al., 2017 |
|  | Melon | 0.88 | Clune et al., 2017 |
|  | Orange | 0.35 | Clune et al., 2017 |
|  | Pear | 0.33 | Clune et al., 2017 |
| Starchy foods |  |  |  |
|  | Bread | 1.55 | Berners Lee et al., 2012 |
|  | Corn | 0.63 | Clune et al., 2017 |
|  | Flour | 1.02 | Vazquez-Rowe et al. 2017 |
|  | Pasta | 0.45 | Röös et al. 2011 |
|  | Potatoes | 0.20 | Clune et al., 2017 |
|  | Rice | 2.66 | Clune et al., 2017 |
| Dairy products |  |  |  |
|  | Greek-style natural yoghurt | 2.92 | Vasilaki et al., 2016 |
|  | Ice cream and other dairy desserts | 2.8 | Berners Lee et al., 2012 |
|  | Milk | 1.23 | González-García et al., 2013 |
|  | Yoghurt | 1.43 | Clune et al., 2017 |
| Herbs and spices and others |  |  |  |
|  | Paprika, parsley | 0.88 | Clune et al., 2017 (form tumeric) |
|  | Salt | 2.55 | Berners Lee et al., 2012 |
|  | Sugar | 0.61 | Menal Puey et al., 2023 |
| Fats/Oil |  |  |  |
|  | Butter | 11.52 | Clune et al., 2017 |
|  | Olive oil | 1.47 | Pattara et al., 2016 |
| Meat and meat derivatives |  |  |  |
|  | Beef | 28.73 | Clune et al., 2017 |
|  | Chicken meat | 2.5 | Gonzalez-García et al. 2014 |
|  | Chicken, whole | 4.12 | Clune et al., 2017 |
|  | Ham | 5.77 | Clune et al., 2017 |
|  | Pork | 5.85 | Clune et al., 2017 |
|  | Sausage | 5.15 | Menal Puey et al., 2023 |
|  | Spicy pork sausage with red pepper (chorizo) | 17.8 | Noya et al, 2016 |
| Prepared or processed foods |  |  |  |
|  | Crips | 1.5 | Berners Lee et al., 2012 |
|  | Croquettes emapanadilas, French fries | 3.5 | Berners Lee et al., 2012 |
| Fish |  |  |  |
|  | Cod | 3.49 | Clune et al., 2017 |
|  | Hake | 8.98 | Clune et al., 2017 |
|  | Pomfret | 6.63 | Clune et al., 2017 |
|  | Trout | 3.73 | Clune et al., 2017 |
|  | Vinegar | 1.55 | Berners Lee et al., 2012 |
| Condiments/Sauces |  |  |  |
|  | Canned tuna fish | 10.23 | Roibas et al., 2018 |
|  | Dehydrated bouillon cube | 7.1 | Milà i Canals et al., 2011 |
|  | Ketchup | 1.6 | Berners Lee et al., 2012 |
|  | Mayonnaise | 1.95 | Hetherington et al., 2012 |
|  | Tomato sauce | 1.03 | Menal Puey et al., 2023 |
| Sugars/Sweets |  |  |  |
|  | Donut | 1.85 | Berners Lee et al., 2012 |
|  | Jam | 3.2 | Berners Lee et al., 2012 |

Note: ^*^References used:

1. Clune S, Crossin E & Verghese K (2017) Systematic review of greenhouse gas emissions for different fresh food categories. *J Clean Prod* **140**, 766–83. doi: 10.1016/j.jclepro.2016.04.082.
2. Berners-Lee M, Hoolohan C, Cammack H *et al.* (2012) The relative greenhouse gas impacts of realistic dietary choices. *Energy Policy* **43**, 184–90. doi: 10.1016/j.enpol.2011.12.054.
3. Vázquez-Rowe I, Larrea-Gallegos G, Villanueva-Rey P *et al.* (2017) Climate change mitigation opportunities based on carbon footprint estimates of dietary patterns in Peru. *PLoS One* **12**, e0188182 . doi: 10.1371/journal.pone.0188182.
4. Röös E, Sundberg C & Hansson PA (2011) Uncertainties in the carbon footprint of refined wheat products: a case study on Swedish pasta. *Int J Life Cycle Assess* **16**, 338–50. doi: 10.1007/s11367-011-0270-1.
5. Vasilaki V, Katsou E, Ponsá S *et al.* (2016) Water and carbon footprint of selected dairy products: A case study in Catalonia. *J Clean Prod* **139**, 504–16. doi: 10.1016/j.jclepro.2016.08.032.
6. González-García S, Castanheira ÉG, Dias AC *et al.* (2013) Using Life Cycle Assessment methodology to assess UHT milk production in Portugal. *Sci Total Environ* **442**, 225–34. doi: 10.1016/j.scitotenv.2012.10.035.
7. Menal Puey S, Lopes IM & Fajó Pascual M (2023) *Informe científico del impacto medioambiental de los alimentos incluidos en los diferentes grupos de las guías dietéticas actuales junto a unas recomendaciones específicas de consumo que tengan en cuenta dicho impacto medioambiental y la salud de la población española para concienciar y sensibilizar a la población.* ECODES. https://www.unizar.es (Accessed 20 November 2024)
8. Pattara C, Salomone R & Cichelli A (2016) Carbon footprint of extra virgin olive oil: A comparative and driver analysis of different production processes in Centre Italy. *J Clean Prod* **127**, 533–47. doi: 10.1016/j.jclepro.2016.03.152.
9. González-García S, Gomez-Fernández Z, Dias AC *et al.* (2014) Life Cycle Assessment of broiler chicken production: A Portuguese case study. *J Clean Prod*. **74**, 125–34. doi: 10.1016/j.jclepro.2014.03.067.
10. Noya I, Aldea X, Gasol CM *et al.* (2016) Carbon and water footprint of pork supply chain in Catalonia: From feed to final products. *J Environ Manage* **171**, 133–43. doi: 10.1016/j.jenvman.2016.01.039.
11. Roibás L, Cuevas A, Vázquez ME *et al.* (2018) Using water scarcity footprint to choose the most suitable location for forest carbon sinks: A case study. *Sustainable Prod Consum* **16**, 1–12. doi: 10.1016/j.spc.2018.06.001.
12. Milà i Canals L, Sim S, García-Suárez T *et al.* (2011) Estimating the greenhouse gas footprint of Knorr. *Int J Life Cycle Assess* **16**, 50–8. doi: 10.1007/s11367-010-0239-5.
13. Hetherington AC, McManus MC & Gray DA (2012) SETAC Europe 18th LCA Case study Symposium: Carbon Footprint Analysis and Life Cycle Assessment of Mayonnaise.

**Supplementary Table 2.** Mean nutrient loss due to food waste and percentage reduction in intake from served to consumed menus.

|  | **Mean nutrient loss due to food waste**  **(per menu, SD)** | **Percentage (%) reduction from served to consumed menus** |
| --- | --- | --- |
| Energy (kcal) | 193.0 (153.3) | 24.4 |
| Total fat (g) | 9.0 (9.0) | 22.7 |
| SFA (g) | 1.8 (2.3) | 19.8 |
| MUFA (g) | 5.6 (6.0) | 23.3 |
| PUFA (g) | 1.1 (1.2) | 22.4 |
| Proteins (g) | 6.6 (6.4) | 22.2 |
| Total carbohydrates (g) | 20.1 (15.2) | 26.9 |
| Simple carbohydrates (g) | 4.6 (4.9) | 24.3 |
| Dietary fibre (g) | 2.8 (2.2) | 31.5 |
| Sodium (mg) | 262.5 (195.4) | 26.8 |
| Vitamin A (µg) | 100.8 (107.0) | 44.0 |
| Thiamine (mg) | 0.1 (0.1) | 25.0 |
| Riboflavin (mg) | 0.1 (0.1) | 22.2 |
| Niacin (mg) | 3.0 (2.9) | 23.1 |
| Vitamin B6 (mg) | 0.2 (0.2) | 25.4 |
| Folate (µg) | 33.4 (31.3) | 31.3 |
| Vitamin B12 (µg) | 0.5 (1.6) | 17.2 |
| Vitamin C (mg) | 17.1 (18.1) | 45.3 |
| Ca (mg) | 38.7 (35.2) | 22.1 |
| I (µg) | 10.3 (20.5) | 26.9 |
| Fe (mg) | 1.3 (1.1) | 27.2 |
| Mg (mg) | 25.5 (20.9) | 26.8 |
| P (mg) | 96.8 (95.7) | 21.9 |
| Zn (mg) | 0.7 (0.8) | 21.2 |

Abbreviations: MUFA, Monounsaturated fatty acids; PUFA, Polyunsaturated fatty acids SD, Standard deviation; SFA, Saturated fatty acids.
